# Supplementary material for: Novel Insights into the Pig Gut Microbiome Using Metagenome-Assembled Genomes
Source: Microbiol Spectr. 2022 Jul 26;10(4):e02380-22. doi: 10.1128/spectrum.02380-22 (PMC9431278; doi:10.1128/spectrum.02380-22)
Supplement: Supplemental file 1 — Supplemental material. Download spectrum.02380-22-s0001.pdf, PDF file, 3.7 MB [file spectrum.02380-22-s0001.pdf]

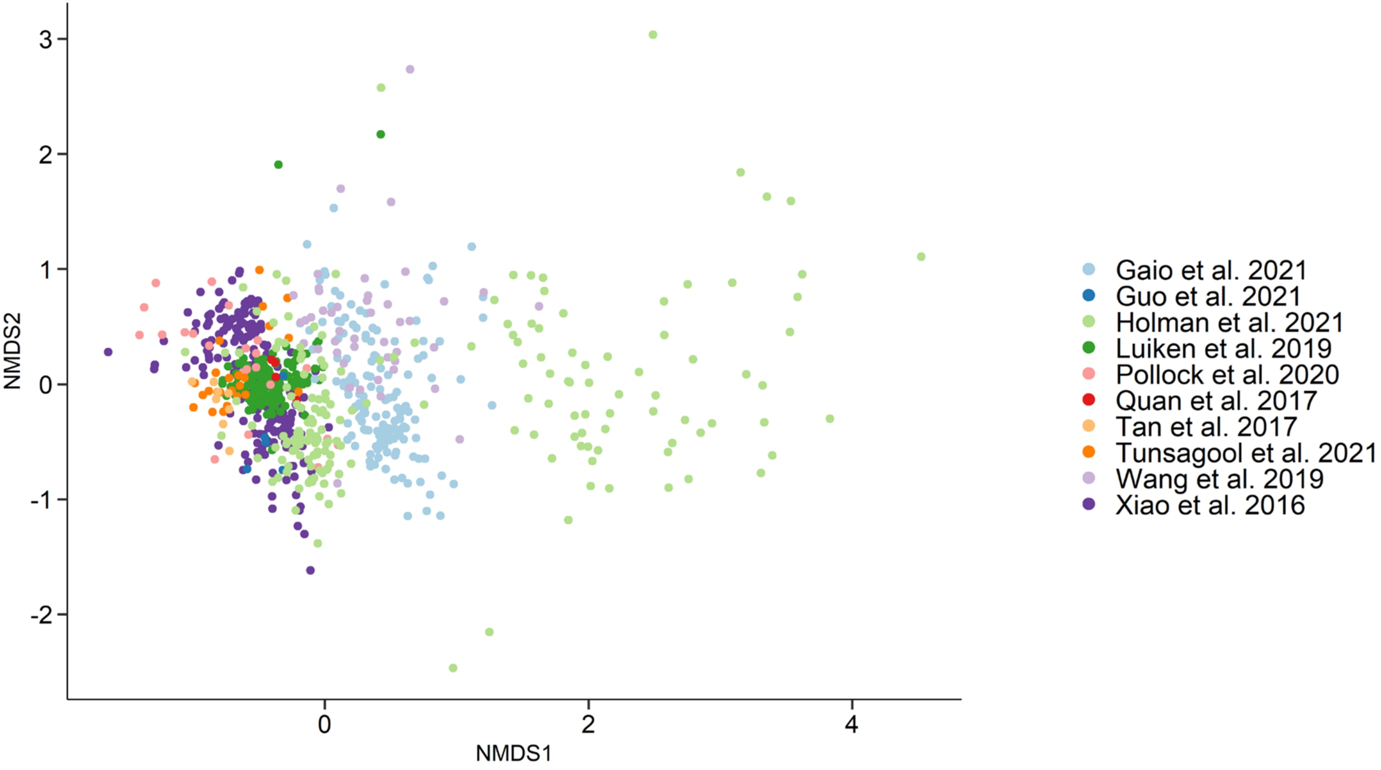

Supplementary Fig. S1. Non-metric multidimensional scaling (NMDS) ordination of the Bray-Curtis dissimilarities for the relative abundance of the 1,150 MAGs in this study and those in 9 publicly available swine metagenomic studies.

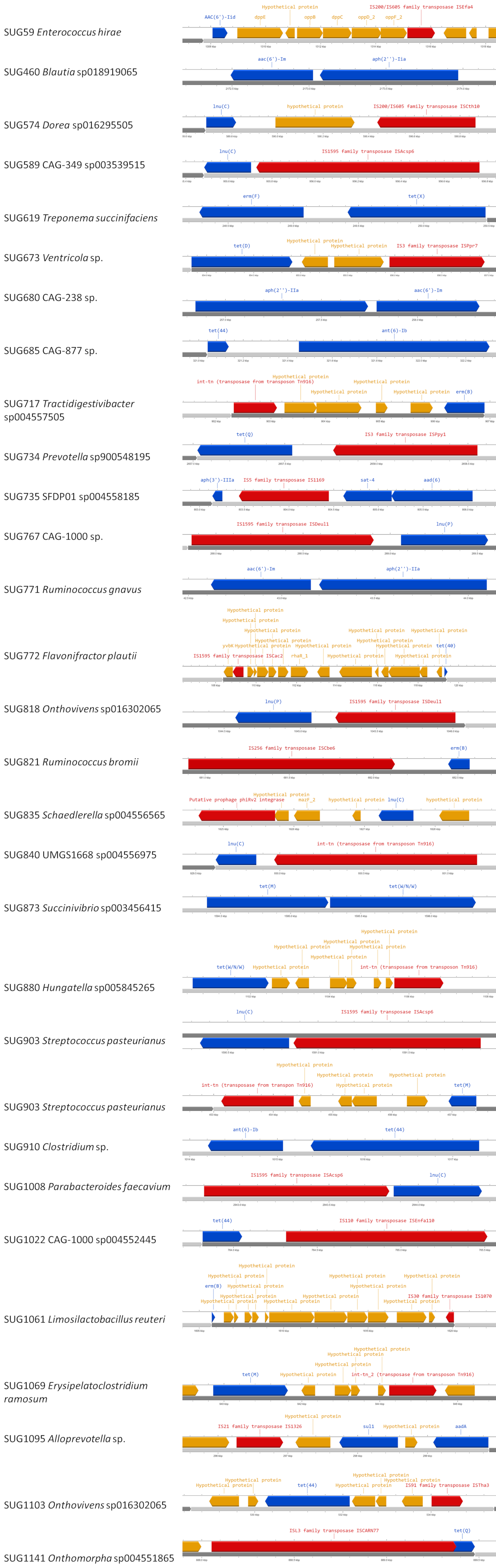

Supplementary Fig. S2. Metagenome-assembled genomes (MAGs) with antimicrobial resistance genes (ARGs) that were co-located on the same contig as other ARGs or with putative transposase or integrase genes.

Supplementary Table S6. Publicly available swine metagenomic samples included in this study.

Study	Country	Number of samples included	Sample type	Instrument	Read length	Accession
(Gaio et al., 2021)	Australia	143	Feces	NovaSeq 6000	2 x 150 bp	PRJNA526405
(Guo et al., 2021)	China	5	Feces	NovaSeq 6000	2 x 150 bp	PRJNA720618
(Munk et al., 2018)	Belgium, Bulgaria, Denmark, France, Germany, Italy, Netherlands, Poland, Spain	171	Feces	HiSeq 3000	2 x 150 bp	PRJEB22062
(Pollock et al., 2020)	United Kingdom	22	Feces	HiSeq 4000	2 x 150 bp	PRJEB34736
(Quan et al., 2019)	China	4	Feces	HiSeq 2500	2 x 150 bp	PRJNA471937
(Tan et al., 2017)	China	8	Cecal contents	HiSeq 2500	2 x 150 bp	PRJNA389749
(Tunsagool et al., 2021)	Thailand	21	Cecal contents	NovaSeq 6000	2 x 150 bp	PRJNA694380
(Wang et al., 2019)	Canada	45	Feces	HiSeq 2500	2 x 125 bp	PRJNA494875
(Xiao et al., 2016)	Denmark, France, China	204	Feces	HiSeq 2000	2 x 100 bp	PRJEB11755

Gaio, D., DeMaere, M.Z., Anantanawat, K., Chapman, T.A., Djordjevic, S.P., and Darling, A.E. (2021) Post-weaning shifts in microbiome composition and metabolism revealed by over 25 000 pig gut metagenome-assembled genomes. *Microb Genom* **7**.

Guo, L., Zhang, D., Fu, S., Zhang, J., Zhang, X., He, J. et al. (2021) Metagenomic Sequencing Analysis of the Effects of Colistin Sulfate on the Pig Gut Microbiome. *Front Vet Sci* **8**: 663820.

Munk, P., Knudsen, B.E., Lukjancenko, O., Duarte, A.S.R., Van Gompel, L., Luiken, R.E.C. et al. (2018) Abundance and diversity of the faecal resistome in slaughter pigs and broilers in nine European countries. *Nat Microbiol* **3**: 898-908.
Pollock, J., Muwonge, A., Hutchings, M.R., Mainda, G., Bronsvoort, B.M., Gally, D.L., and Corbishley, A. (2020) Resistance to change: AMR gene dynamics on a commercial pig farm with high antimicrobial usage. *Sci Rep* **10**: 1708.
Quan, J., Cai, G., Yang, M., Zeng, Z., Ding, R., Wang, X. et al. (2019) Exploring the Fecal Microbial Composition and Metagenomic Functional Capacities Associated With Feed Efficiency in Commercial DLY Pigs. *Front Microbiol* **10**: 52.
Tan, Z., Yang, T., Wang, Y., Xing, K., Zhang, F., Zhao, X. et al. (2017) Metagenomic Analysis of Cecal Microbiome Identified Microbiota and Functional Capacities Associated with Feed Efficiency in Landrace Finishing Pigs. *Front Microbiol* **8**: 1546.
Tunsagool, P., Mhuantong, W., Tangphatsornruang, S., Am-In, N., Chuanchuen, R., Luangtongkum, T., and Suriyaphol, G. (2021) Metagenomics of antimicrobial and heavy metal resistance in the cecal microbiome of fattening pigs raised without antibiotics. *Appl Environ Microbiol* **87**: e02684-02620.
Wang, W., Hu, H., Zijlstra, R.T., Zheng, J., and Ganzle, M.G. (2019) Metagenomic reconstructions of gut microbial metabolism in weanling pigs. *Microbiome* **7**: 48.
Xiao, L., Estelle, J., Kiilerich, P., Ramayo-Caldas, Y., Xia, Z., Feng, Q. et al. (2016) A reference gene catalogue of the pig gut microbiome. *Nat Microbiol* **1**: 16161.
